# Low Bone Mineral Density in Hemophiliacs

**DOI:** 10.3389/fmed.2022.794456

**Published:** 2022-02-02

**Authors:** Jennifer Gebetsberger, Michael Schirmer, Walter J. Wurzer, Werner Streif

**Affiliations:** ^1^Department of Pediatrics I, Medical University of Innsbruck, Innsbruck, Austria; ^2^Department of Internal Medicine, Clinic II, Medical University of Innsbruck, Innsbruck, Austria; ^3^Speciality Pharma Service Austria, Altenmarkt, Austria

**Keywords:** coagulation, hemophilia A, factor VIII, bone mineral density, osteopenia, osteoporosis

## Abstract

**Objective:**

To review the current knowledge on bone health in patients with hemophilia A and the underlying pathogenetic mechanisms.

**Data Sources:**

Original research articles, meta-analyses, and scientific reviews.

**Data Synthesis:**

Already in childhood, patients with hemophilia A are prone to low bone mineral density, leading to osteopenia and/or osteoporosis. Initially associated with the life style of hemophilia, today we are faced with accumulating evidence that coagulation factor VIII is involved directly or indirectly in bone physiology.

**Conclusion:**

Understanding the role of factor VIII and the mechanisms of decreased bone mineral density in hemophilia A is critically important, especially as non-factor replacement therapies are available, and treatment decisions potentially impact bone health.

## Introduction

Prognosis and life expectancy for patients with hemophilia A have dramatically improved over the last decades. With prophylactic treatment, patients can prevent major bleeding events and live an almost normal life. However, there is growing evidence that the lack of coagulation factor VIII (FVIII) also impacts bone health, leading to decreased bone mineral density (BMD). Since factor replacement therapies are able to improve the bone phenotype and given the increasing use of non-replacement therapies in patients with severe hemophilia, the scientific community is prompted to investigate whether FVIII plays important roles outside the coagulation system.

This review provides a summary of recent literature on bone modeling in hemophilia A and sheds light on the underlying pathophysiological mechanisms. It thus raises questions that remain to be answered in future studies.

## The Coagulation System

The coagulation system entails a highly regulated cascade that ultimately leads to hemostasis, which is the blocking of bleeding. Primary hemostasis is characterized by the activation, aggregation and adherence of platelets at the site of vascular injury exposing subendothelial collagen and von Willebrand factor (vWF). However, since platelets and vWF are not enough to form a stable thrombus at the site of vessel injury, secondary hemostasis steps in to form a clot at the site of injury. This clot formation depends on several substances called clotting factors, which activate each other in what is known as the clotting cascade (1).

**Figure 1:**
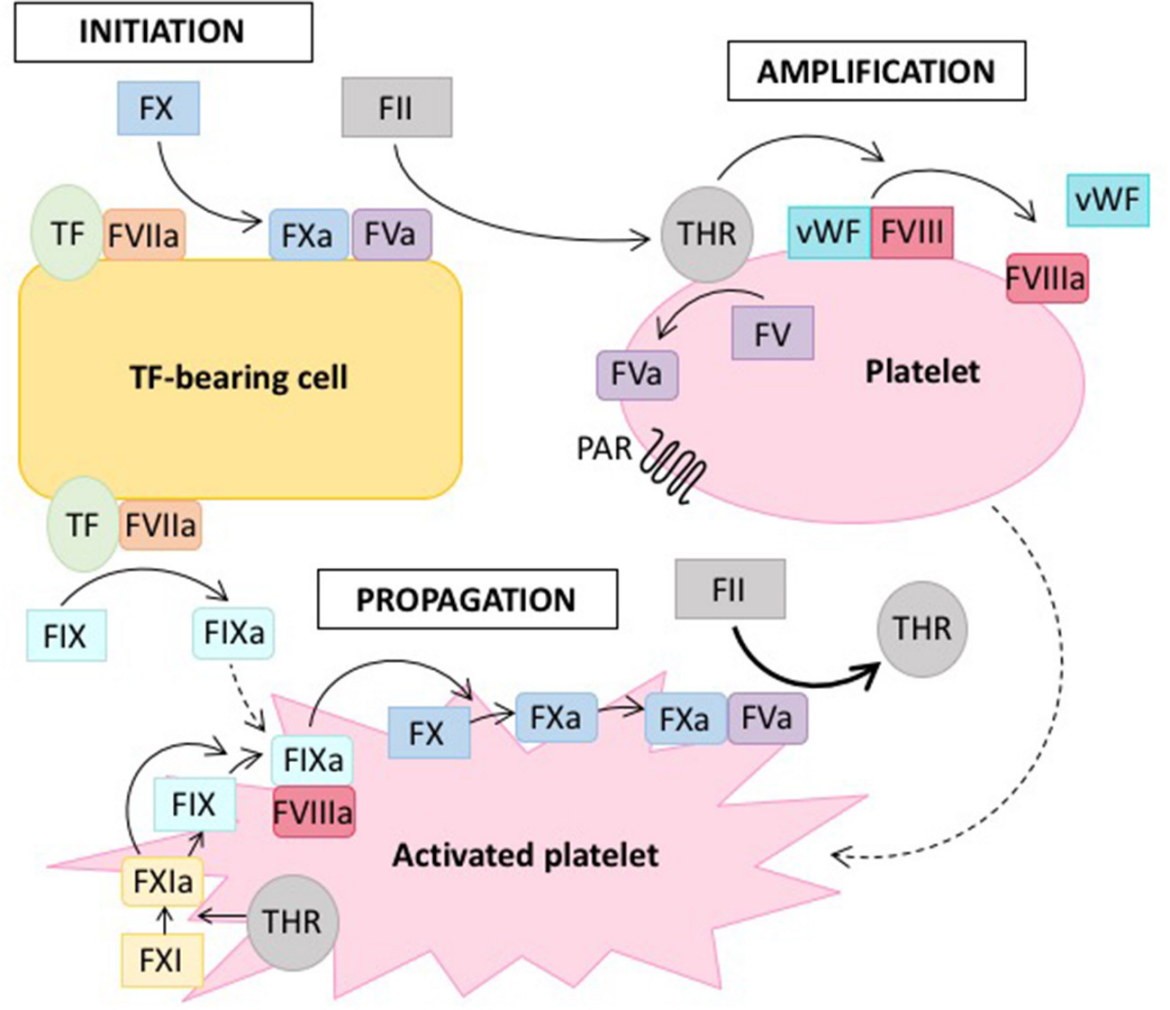
Cell-based model of coagulation. On the surface of tissue factor (TF) -bearing cells, coagulation is initiated by TF, which leads to the generation of a small amount of thrombin (THR) from prothrombin (FII) (initiation phase). Thrombin amplifies the initiation signal by activating platelets via protease-activated receptors (PAR) and cofactors (FV, FVIII) on the platelet surface (amplification or priming phase). Large amounts of thrombin are then generated on the surface of activated platelets (propagation phase). Solid lines indicate proteolytic conversion of an inactive zymogen to its active form, dotted lines indicate activation. Adapted from reference (7).

Several models of this clotting cascade have emerged in the literature. The classical model from 1964 generally describes two separate pathways, the intrinsic and the extrinsic one, which converge in the final common pathway to ultimately form fibrin (2). However, this model slowly evolved toward a cell-based model (see Figure 1) (3), which allowed the integration of several clinical observations that were not consistent with the classical model of coagulation (4, 5). Whereas, the classical model describes a rather unidirectional proteolytic cascade, the cell-based model suggests that *in vivo* thrombus formation actually occurs in three overlapping phases: initiation, amplification and propagation (see Figure 1) (3, 4, 6).

## Hemophilia

Hemophilia is a X-chromosomal recessive bleeding disorder caused by dysfunction of the intrinsic tenase complex, which consists of coagulation factors VIIIa and IXa (see Figure 1). Hemophilia caused by mutations in the *F8* gene is referred to as hemophilia A and the disease linked to defects of the *F9* gene is termed hemophilia B (8). Hemophilia A is the more common form, affecting ~1 out of 5.000 live male births, whereas hemophilia B occurs in 1 out of 30.000. The clinical presentation of hemophilia A and B are similar. Severe cases with factor activity below one percent are characterized by spontaneous bleeding, whereas in moderate to mild cases bleeding occurs trauma-associated, albeit under milder circumstances than normal (9). Hemophilia is treated by infusion of the deficient factor either prophylactically or on-demand, whereas also other novel therapeutic strategies aim at rebalancing the coagulation system in the absence of FVIII (10).

## Low Bone Mineral Density in Hemophiliacs

The development and wider availability of factor concentrates have led to a dramatic increase in the life expectancy of patients with hemophilia (11). Concomitantly, comorbidities associated with hemophilia were recognized or became more pronounced as a result of patient aging (12). While joint damage (arthropathy), as a consequence of recurrent bleeding into joints (13), is certainly the primary co-morbidity of hemophilia, research conducted in the last 30 years has indicated that hemophiliacs are at an increased risk for low bone mineral density (BMD) (14). In a study of young adults (median age 41.5 years), as many as 70% of patients with hemophilia exhibited decreased BMD, with 43% displaying osteopenia and 27% osteoporosis (15). Although the risk of hemophilia-related osteoporotic fractures has not been well-established, there are a few studies showing a higher fracture incidence in hemophilic subjects compared to the control population (16–19).

In patients with hemophilia, BMD was first evaluated by Gallacher and colleagues after they observed a non-traumatic lumbar compression fracture in a 31-year-old man and a fracture of the femoral neck after an epileptic seizure in a 20-year-old man, both of them having severe hemophilia A (20). Subsequently, they evaluated another 19 adults with severe hemophilia and compared their BMD in the lumbar spine and the femoral neck with the BMD of age-matched controls. The mean spine and femoral neck BMD was significantly lower in those with hemophilia than in controls (0.13 and 0.19 g/cm^2^, respectively). Several studies followed and showed similar, although less dramatic findings (19, 21–23).

However, two meta-analyses confirmed the link between hemophilia and secondary osteoporosis (24, 25). Both analyzed case-control studies that used dual energy x-ray absorptiometry (DEXA) to investigate BMD of the lumbar spine in patients with hemophilia as the primary outcome. The analysis by Iorio et al. covered seven studies involving 101 cases and 307 age- and gender-matched controls, including both pediatric and adult hemophiliacs (24). Their analysis confirmed a significant reduction in lumbar spine BMD in severe hemophilia patients as compared to controls, both in adults and children (24). The study by Paschou and co-workers looked at all the studies included in the previous meta-analysis and added another six published before 2012 (25). Their meta-analysis covered 415 patients and 585 age- and gender-matched controls and also reported BMD of the lumbar spine to be significantly lower in cases than in controls. Additionally, they delineated significantly reduced BMD of the femoral neck and an insignificant reduction in total hip BMD (25). Several more recent case-control studies also reported reduced BMD at these two skeletal sites, but did not find any significant differences in the lumbar spine (23, 26–31).

Taken together, the skeletal area most prone to loss of BMD, the etiology, and the underlying pathogenetic mechanisms are still subjects of debate. The data strongly support the finding that hemophiliacs are at increased risk to develop secondary osteoporosis as compared to the general population, with a higher prevalence of low BMD already in children (24, 32–37). However, the results of several studies also indicate that the use of prophylactic factor treatment since early childhood may preserve normal BMD in severe hemophilia (38, 39).

The central question posed by this evidence is whether the low BMD in hemophilia is caused directly by the coagulation defect or whether it develops secondary to either a comorbidity or the life style that affected patients are forced to accept.

## Risk Factors for Low Bone Mineral Density in Hemophilia

The main risk factors predisposing to reduced bone density include smoking, alcoholism, vitamin D deficiency, and some drugs such as exogenous glucocorticoid excess, anticoagulants of warfarin, and heparin (40).

One of the main risk factors in hemophilia discussed as an underlying cause of low BMD and as a factor facilitating the prevention of osteoporosis is physical activity (41–43). The fear of trauma and bleeding, as well as hemophilic arthropathy (HA) typically associated with chronic pain and structural changes in the joints, lead to inactivity and lack of weight-bearing exercise (44). This may compromise the development of peak bone mass already in childhood and consequently affects BMD in adult life. While a lower level of physical activity seems a feasible explanation, some studies have suggested that it alone does not account for the decrease in BMD (45, 46).

Additionally, it has often been argued that osteoporosis and osteopenia in hemophiliacs can be simply explained by HA, the repeated bleeding in the joints, *per se* (47, 48). If this was true, the frequency and or severity of these comorbidities should not be elevated in hemophiliacs who are on primary prophylaxis with no or only minimal joint bleeds. Khawaji et al. suggested that long-term factor replacement in severe hemophilia indeed tends to preserve BMD (49), but others failed to replicate the data (50).

Other risk factors for low BMD, that are often present in the hemophilia population, include low vitamin D levels, low body mass index (BMI), and blood-borne virus infections (15). There is a strong prevalence of hypovitaminosis D in hemophiliacs (51, 52). Vitamin D plays a pivotal role in bone mineralization, where it promotes calcium absorption from the gut (53). However, most of the studies found a correlation with BMD only in children (33, 39, 54), but not in adults (28). Moreover, the meta-analysis by Iorio and colleagues (24), as well as other subsequent studies, found no correlation between low BMI, or hepatitis C/HIV infections, and low BMD (28, 55).

Over the last few years, however, striking clinical and experimental evidence has shown that FVIII deficiency leads to decreased BMD independently of the aforementioned risk factors (56). As such, mice genetically engineered to be deficient in FVIII show a loss of BMD as compared with wild type controls, despite the fact that they have the same activity level, no increased hemarthroses and are not affected by other comorbidities (57–59). These experimental data suggest that FVIII may play a role outside the coagulation system, directly or indirectly affecting bone metabolism.

## Bone Metabolism

Before drawing connections between the coagulation system and bone mineral density, it appears important to provide an insight into how bone metabolism functions in human beings.

**Figure 2:**
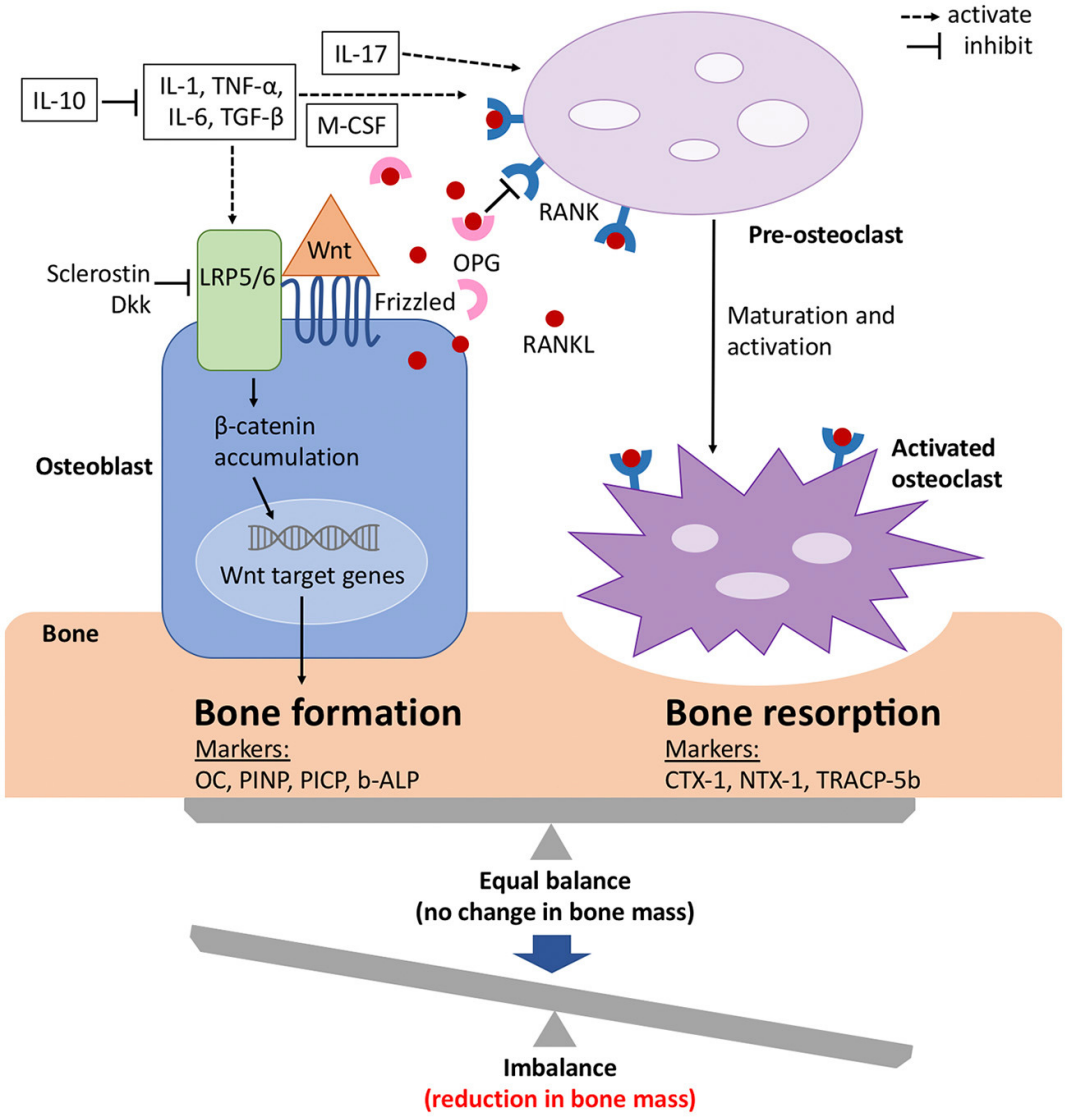
Signaling mechanisms with balance of bone formation and resorption in bone metabolism. The balance between bone formation and bone resorption is largely regulated by the Wnt/ β catenin pathway and the RANK/RANKL/OPG axis. Wnt protein binds to its co-receptors Frizzled and LRP5/6 on the surface of osteoblasts, which leads to the stabilization and accumulation of β-catenin in the cytoplasm, before it translocates to the nucleus where it regulates target genes and induces bone formation. This pathway can be inhibited by sclerostin and members of the Dkk protein family. RANKL binds to its receptor RANK, which is expressed on pre-osteoclasts. This induces the activation of several transcription factors and in turn osteoclast differentiation and maturation. OPG, which is a soluble decoy receptor and also secreted by osteoblasts, can bind to RANKL and thereby inhibits bone resorption. Under physiological conditions, OPG and RANKL are in equilibrium and preserve bone homeostasis. Several proinflammatory cytokines and growth factors (white boxes) stimulate and upregulate RANKL expression and mediate osteoclast maturation and activation. The metabolic state of the bone is reflected by its biochemical products, which also serve as bone turnover markers for either bone formation or resorption. b-ALP, bone-specific alkaline phosphatase; CTX-1, carboxy-terminal cross-linking telopeptides of type I collagen; Dkk, Dickkopf; IL, interleukin; LRP, low-density lipoprotein receptor-related protein; M-CSF, macrophage colony-stimulating factor; NTX-1, amino-terminal cross-linking telopeptides of type I collagen; OC, osteocalcin; OPG, osteoprotegerin; PICP, procollagen type 1 carboxy-terminal propeptide; PINP, procollagen type 1 amino-terminal propeptide; RANK, receptor activator of nuclear factor-kappa B; RANKL, receptor activator of nuclear factor-kappa B ligand; TGF-β, transforming growth factor β; TNF-α, tumor necrosis factor α; TRACP-5b, tartrate-resistant acid phosphatase 5b; Wnt, wingless related integration site. Adapted from references (61, 62).

Although bone has an inert appearance, it is a dynamic tissue that is continuously resorbed by osteoclasts and formed anew by osteoblasts (see Figure 2). These processes are tightly coupled, so that under normal conditions new bone formation cannot occur without antecedent bone resorption. However, when these processes are out of balance, bone loss occurs (Figure 2) (60).

Several peptides, cytokines and growth factors are produced by osteoblasts, osteoclasts and osteocytes (main cell population of the skeletally mature bone), which facilitate communication and balanced activity. The two pathways that are especially important for bone physiology are the RANK/RANKL/OPG axis and Wnt/β catenin signaling, with the former regulating osteoclast formation and the latter osteoblast differentiation (63).Osteocytes thereby mainly act as orchestrators since they produce factors that influence the activity of both osteoblasts and osteoclasts (64, 65).

The receptor activator of nuclear factor kappa-B (RANK) is a member of the tumor necrosis factor (TNF) receptor family and is expressed on the cell surface of osteoclast precursors. RANK ligand (RANKL) is a transmembrane ligand mainly expressed on osteoblasts/stromal cells in the bone environment (Figure 2). Upon binding of RANKL to its receptor RANK osteoclast proliferation and differentiation are activated. Osteoprotegrin (OPG), another member of the TNF receptor superfamily, competes for binding of RANKL to RANK and thus serves as a decoy receptor for RANKL (Figure 2) (66). By means of this mechanism, RANKL inhibits, whereas OPG promotes, osteoclast apoptosis and any change in this balance leads to pathophysiological conditions (67).

In recent years, also Wnt/β catenin signaling has gained considerable attention with regard to bone metabolism. When the canonical Wnt ligands are present, they bind to the frizzled receptors and one of the co-receptors, either low-density lipoprotein receptor-related protein 5 (LRP5) or 6 (LRP6) (Figure 2) (68, 69). This induces the stabilization and accumulation of cytosolic β catenin, which subsequently translocates to the nucleus where it initiates the transcription of Wnt target genes (70). Sclerostin, as well as members of the Dickkopf (Dkk) protein family, decreases osteoblastic bone formation by acting as antagonists of the Wnt/β catenin pathway by binding to LRP5 and LRP6 on the cell membrane of osteoblasts (Figure 2) (71–74). The function of the entire signaling pathway is very complex, which is reflected by the fact that genetic mutations of single protein members at different stages of osteoblast differentiation lead to different phenotypes (63).

In addition to the aforementioned pathways, cytokines are also known to play key roles in bone metabolism (Figure 2) (75). To name only a few, tumor necrosis factor (TNF) α, for example, has paradoxical effects on bone metabolism, as it activates differentiation of both osteoclasts and osteoblasts and plays roles in various disease states. Interleukin (IL) −1α, IL-1β, IL-6 and macrophage colony-stimulating factor (M-CSF) positively regulate bone resorption by osteoclasts, whereas IL-10 inhibits osteoclastogenesis by means of several distinct mechanisms (76, 77).

For assessment of the state of bone metabolism, several biomarkers that are associated with specific bone processes and overall skeletal health have gained attention. These bone turnover markers (BTM) include biochemical products that represent either bone formation or bone resorption (Figure 2) (60, 78, 79).

## Bone Turnover Markers in Patients With Hemophilia

A few clinical studies have assessed BTMs in hemophiliacs to gain insights into why mineral bone density is diminished and whether the osteoblastic activity is decreased or osteoclastic activity is increased (see Figure 2). The obtained data are controversial: In a study of children with hemophilia A, low levels of osteocalcin, a serum marker of bone formation (Figure 2), were measured but no difference in bone resorption was detected as compared to healthy controls (80). In contrast, the analysis of BTMs in two other studies in children with severe hemophilia A indicated decreased osteoblastic activity as well as increased osteoclast-mediated resorption (81, 82). This stands again in contrast to the results of a study in adults with hemophilia, in which increased levels of NTX-1, CTX-1, and TRACP-5b pointed to up-regulated osteoclastic activity (Figure 2) that was not accompanied by a compensatory increase in osteoblastic activity (83). Three other studies also reported elevated CTX-1 levels in the serum of hemophilia A patients (79, 84, 85). However, another study detected only significantly elevated b-ALP concentrations in male hemophilia patients with low BMD, whereas osteocalcin, NTX-1, CTX-1, and TRACP-5b did not change significantly as compared to patients with normal BMD (78). These inconsistent data are difficult to confirm and may be explained by the complexity of the disease, since other, already mentioned risk factors for low BMD may be present but unequally distributed in the study populations. Therefore, several investigators have analyzed BTM in a hemophilic mouse model. FVIII knockout mice are already well-established and show the same bone phenotype as humans, as they fail to achieve peak bone mass (57–59). Animals at 20 weeks of age did not show any significant difference in the biochemical markers of bone formation or osteoclastogenesis (57). However, another research group detected diminished bone formation in male FVIII knockout mice at 6 months of age, indicated by increased PINP values (86), as also found in a clinical study of 35 male patients with severe hemophilia A and B (87). Cell culture experiments also point to either a qualitative or a quantitative defect in bone formation, since cells isolated from the marrow of FVIII-deficient mice and cultured to induce osteoblasts showed defects in growth, differentiation, and mineralization (88, 89).

## The Role of FVIII in Bone Health

**Figure 3:**
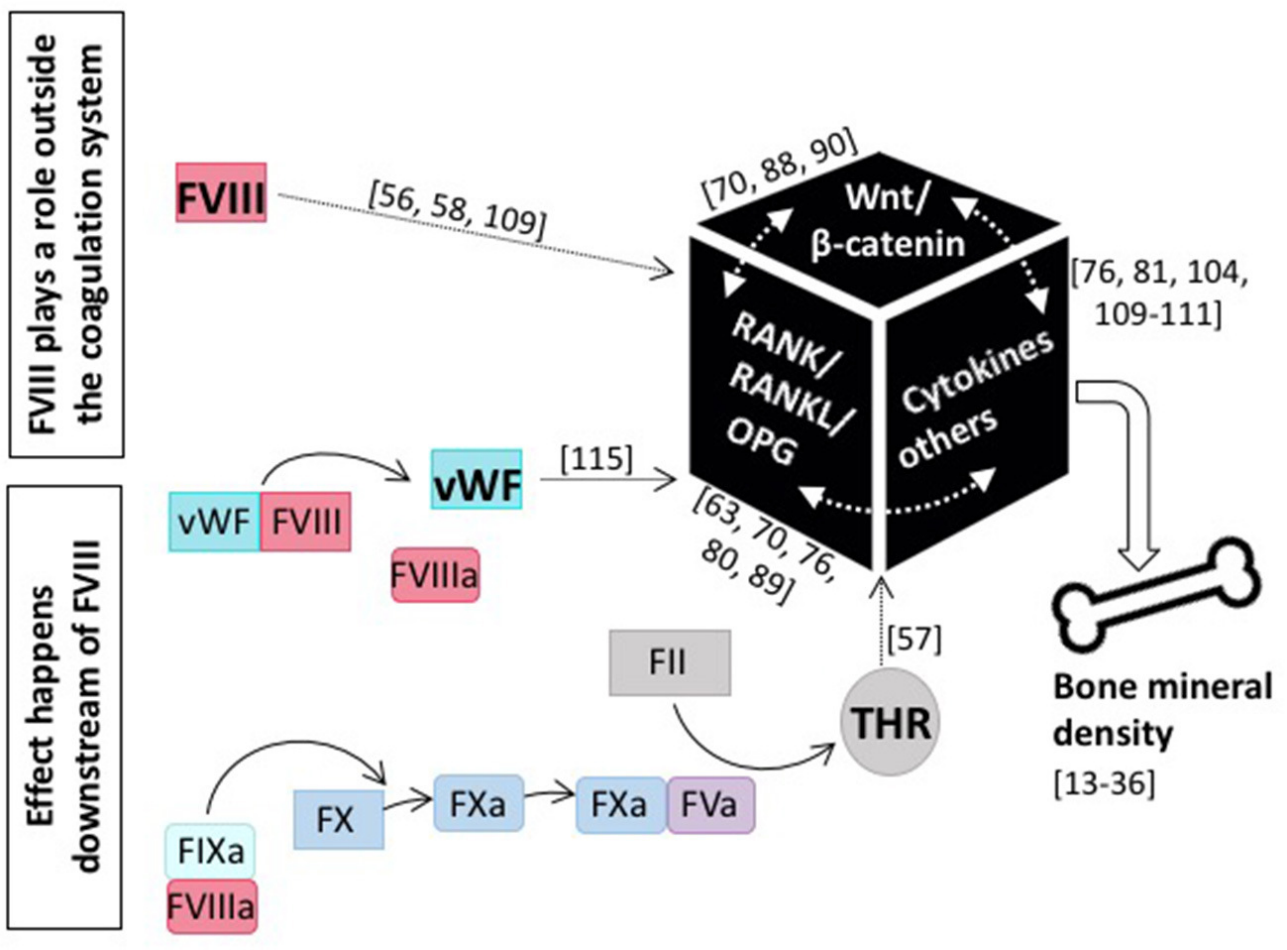
The role of coagulation FVIII in bone health. Current knowledge tells us that several modes of action for FVIII impacting bone mineral density are feasible: either FVIII plays a role outside the coagulation system and directly interacts with main players of bone physiology, including the RANK/RANKL/OPG axis and/or the Wnt/β-catenin pathway, as well as pro-inflammatory cytokines, or the effect happens downstream of FVIII and e.g., the missing interaction with vWF or decreased thrombin production is causative. References for original publications and further reading are written in brackets.

Although the assessment of BTM does not clearly delineate whether decreased bone formation or increased bone resorption is the main cause of the observed reduction in BMD in patients with hemophilia, it is obvious that it is intrinsic to FVIII deficiency. This is underscored by the finding that FVIII replacement is able to reverse the bone phenotype (45, 79, 84, 90). This of course raises the question as to the role of FVIII in bone health (see Figure 3). Does it play a role outside the coagulation system, probably by interfering with the RANK/RANKL/OPG axis or the Wnt/β catenin pathway, or does it affect bone health downstream of FVIII, and is the occurrence of inflammation, the defect in hemostasis, or some other more global phenomenon causative?

### RANK/RANKL/OPG

The role of the RANK/RANKL/OPG signaling pathway in the development of low BMD in patients with hemophilia has interested several research groups. Again, the data are very controversial. In a mouse study, no statistical difference in RANKL or OPG serum levels or the RANKL/OPG ratio between the FVIII KO mice as compared with WT controls at 20 weeks of age was found (57). The same result was obtained in a study of boys (mean age 10.11 ± 6.1 years) (91), and two studies with adults suffering from hemophilia A (79, 83). However, another study in young men (average age 12 years) found increased RANKL and decreased OPG levels (92). Similarly, a study of male adults (mean age 45.9 ± 15.3 years) with hemophilia, found higher serum levels or RANKL and RANKL/OPG ratio than in controls, but patients with low BMD had higher OPG concentrations than did those with normal BMD (73). Interestingly, one study analyzed the RANKL and OPG levels not only in the serum of patients with hemophilia, but also in the synovial tissue of hemophilic joints (66) and found that the serum levels of RANKL and OPG were lower than in healthy controls, whereas at the tissue level increased expression of RANK and RANKL and decreased expression of OPG were detected (66). This raises the question as to how accurately alterations in serum markers of the RANK/RANKL/OPG system reflect local changes in bone turnover.

### Wnt/β Catenin Pathway

Only few data are available on the Wnt/β catenin pathway and its inhibitors in hemophilia patients. Two studies in children found a significant elevation of serum sclerostin in the hemophilic group as compared to the age-matched control group (91, 93). The authors speculated that this elevation may identify hemophilic patients at high risk for developing a bone phenotype (93). Importantly, no additional significant differences in OPG, RANKL or Dkk-1 levels were observed (91). This stands in contrast to data from adult patients with hemophilia, in which lower serum sclerostin and Dkk-1 levels were found. Here, the levels of sclerostin even correlated with the severity of the disease, with patients with severe hemophilia having lower sclerostin concentrations than those with mild or moderate disease (73). However, this increased osteoblastic activity was accompanied by increased osteoclastic activity, which was indicated by elevated levels of RANKL (73).

### Cytokines

As already mentioned, arthropathy as a result of recurrent joint bleeding is the primary co-morbidity of hemophilia (13, 94, 95). There is growing evidence to show that intra-articular inflammation and angiogenesis are pivotal processes in the pathogenic cascade of HA (96). Thereby joint bleeding favors iron release from hemoglobin, thus indicating a chronic inflammatory reaction mediated by cytokines and pro-angiogenic factors, which in turn enhance articular cartilage and subchondral bone destruction (97–100). In skeletally mature FVIII-deficient mice, induced knee joint hemorrhage caused a 25–30% decrease in trabecular bone density already 2 weeks after injury (90). Additionally, HA has been associated with reduced BMD in several studies using multivariable analysis (83, 101). It is already well-known that chronic inflammatory diseases can lead to bone loss, as known from psoriasis, ankylosing spondylitis, systemic lupus erythematosus, multiple sclerosis, inflammatory bowel disease, rheumatoid arthritis (RA), and other disorders (102–105). HA shows similarities to the inflammatory state of RA, but also to the degenerative character of osteoarthritis (OA) (66, 106). However, in comparison to RA and OA, the synovium of patients with hemophilia showed comparable (99), or even the highest concentration of IL-1, IL-6 and TNF-α measured (107). Increased levels of the pro-inflammatory cytokines IL-1β, IL-6, keratinocyte-derived chemokine (KC) and monocyte chemotactic protein-1 (MCP-1) were also detected in synovial fluid of hemophilia A mice with experimentally induced joint hemorrhage (108). Several studies support the connection between those inflammatory cytokines and osteoclast activity (75, 105, 109–112). However, two experimental studies in hemophilic mice reported by Liel et al. showed that FVIII deficiency led to decreased BMD independently of HA (59, 113). Interestingly, these mice had decreased serum levels of IL-1α and IFN-β as compared to wildtype controls (79, 113). Similar results were obtained in 79 patients with severe hemophilia A, where significantly lower serum levels of TNF-α, IL-10 and IL-12 were found in comparison to age-matched healthy controls (84), thus giving rise to the possibility that FVIII deficiency leads to decreased bone-associated cytokine production. However, it might be important to note that the study participants all received FVIII substitutions, either on-demand or prophylactic (84). When the immunological profile of untreated patients with hemophilia A was assessed in comparison with the profile of healthy controls, higher levels of IL-4, IL-6, IL-8, IL-10, IL-12 and IL-17 were detected (114). However, these inconsistencies may also indicate that serum levels of cytokines do not accurately reflect what is happening in the local bone environment or that there exist natural antagonists, which may interfere with data interpretation. As such, when the levels of inflammatory cytokines were measured in the synovial fluid of hemophilic mice, IL-1β, IL-6 and TNF-α were significantly increased but were below the detection limit in the serum from whole blood samples (115).

Altogether, the collected data are difficult to interpret and further studies are urgently needed to clarify whether altered cytokine profiles and/or inflammation are an epiphenomenon of FVIII deficiency or cause and effect for low BMD.

### Missing Interaction With vWF

To be protected from proteolytic degradation, FVIII is bound to vWF in circulation (Figure 1) (116). At sites of vascular damage, adhesion of vWF not only initiates plug formation, but also brings FVIII in proximity to promote the generation of thrombin and fibrin (117, 118). *In vitro* studies further demonstrated that vWF in complex with FVIII is able to inhibit RANKL-induced bone resorption and enhances the inhibitory effect of OPG (96). The FVIII-vWF complex was able to bind to RANKL, whereas each factor alone was not. Thus, the authors suggested that the low BMD in hemophilia is due to increased osteoclastogenesis caused by deficient FVIII-vWF complex (96). However, Taves et al. demonstrated that FVIII- and FIX-deficient mice, but not vWF-deficient mice, developmentally show an osteoporotic phenotype, indicating that the FVIII-vWF complex is not necessary for normal bone physiology *in vivo* (119). Furthermore, a clinical association between low BMD and von Willebrand disease, which would be caused by a defect or deficiency of vWF, has so far not been reported.

### Decreased Thrombin Production

Deficiency of FVIII as well as FIX results in the inhibition of FX activation and thus in failed thrombin production (Figure 1) (120). As already mentioned above, animal studies with FVIII and FIX knockout mice point toward a similar bone phenotype (119, 121, 122), indicating that thrombin generation and signaling are important for bone health. Indeed, it was shown that thrombin plays a role in bone remodeling as it is able to cleave osteopontin, which is important for anchoring osteoclasts to the mineralized matrix (123). Furthermore, thrombin has been found to inhibit osteoclast differentiation and to down-regulate the expression of RANK in isolated pre-osteoclast cultures (124). Additionally, thrombin stimulates osteoblast proliferation and inhibits osteoblast differentiation and apoptosis (125–127). Some of the described effects are believed to be mediated by the expression of growth factors and cytokines (128). This might explain the low serum cytokine levels in hemophilia A patients, as described above (113). Many but not all cellular responses to thrombin are mediated by the three thrombin receptors PAR-1, PAR-3, and PAR-4 (128–130). Importantly, PAR-1 has gained most attention in this context, since it is expressed in osteoblasts (but not osteoclasts), whereas PAR-3 is not expressed in murine osteoblasts and PAR-4 has only been reported in osteoblast-like calvarial cells (129, 131). Aronovitch and colleagues hypothesized that an absence of FVIII leads to deficient thrombin production, which results in ineffective thrombin-mediated signaling by PAR-1 receptors and abnormal bone physiology (58). In support of their hypothesis they showed bone phenotypes in PAR-1 similar to those in FVIII-deficient mice (58). Contrarily, Tudpor and colleagues found increased BMD in PAR-1 KO mice, which was accompanied by a decreased RANKL/OPG ratio (132). The assumption that PAR-1 alone cannot be responsible for low BMD in hemophilia is also substantiated by the finding that thrombin inhibited osteoclast differentiation in different organs in PAR-1 KO mice (124). Additionally, a >85% prothrombin KO in 3-week-old wild-type mice did not significantly impact bone health (133). These data suggest that either thrombin deficiency is not decisively responsible for low BMD in hemophilia, or the impact occurs already very early in bone development.

## Conclusion and Future Directions

Patients with hemophilia are prone to develop low BMD. Whether this bone phenotype is intrinsic to FVIII deficiency or secondary to it, is the subject of debate. Several research groups have speculated that FVIII plays a role outside the coagulation system, likely by directly interfering with the RANK/RANKL/OPG axis and/or the Wnt/β pathway, or indirectly via modulating cytokines and/or other factors (Figure 3). Since also FIX deficiency results in a similar bone phenotype (22, 51), it is also plausible that the effect takes place further downstream in the coagulation cascade. However, research so far indicates that there is not one and only one adjustment screw and the cause for low BMD is instead multifactorial. Things are even more complicated by the fact that there exist several interconnections between the individual pathways and bone physiology is rather complex in nature. Thus, the underlying pathogenic mechanism may be multifactorial with reduced bone formation occurring due to disordered bone metabolism secondary to factor deficiency and increased bone resorption occurring due to joint bleeding, lack of adequate weight-bearing exercise and inflammation with the relative contribution of each mechanism changing over a patient's lifetime (134).

Unfortunately, there is still a large knowledge gap that is mainly due to discrepancies in data that are difficult to interpret. As genetics plays a critical role in bone mass and turnover, differences in mouse strains used in the animal models may contribute to different outcomes. Also, other variables such as microbiota differences between animal vendors and institutional housing facilities can have significant effects in mouse studies (135). Additionally, partial synthesis of defective FVIII proteins in comparison to a total KO, irrespective of functionality, may advocate an inflammatory state that additionally, or erroneously, stimulates bone resorption. It becomes even more difficult to determine causal roles for FVIII or other coagulation factors in clinical studies since they can be affected by a complex interplay of confounding factors, such as viral infections, medication, hypovitaminosis D, smoking, alcoholism, and many more. Furthermore, pathophysiological changes in other organs or cells, such as cardiovascular cells, kidneys, and macrophages, can impact bone biology (136–138). More clinical and experimental studies are thus urgently needed. However, both require more comprehensive indices, especially those generated by proteomic and genomic assays. One of the main challenges with comparison and interpretation of clinical studies is the lack of uniformity of definitions of events or end points (139). The Joint Health Markers *MOTIVATE* sub-study thus aims to evaluate the impact of different treatment approaches on levels of established bone health markers and to identify and validate new markers of early-onset bone damage (140).

Clinical studies should more often also address patients with factor inhibitors, since the literature shows that these have hardly been considered. Some of the immune tolerance induction (ITI) protocols use one or more immunosuppressive agents for the eradication of inhibitors associated with the side-effect of reduced BMD, thus further pushing patients toward osteoporosis (40). It will also be interesting to see if patients treated with emicizumab, a recombinant, humanized, bispecific monoclonal antibody that restores the function of FVIII by bridging activated FIX and FX, develop a bone phenotype or not. Data from the HAVEN3 study so far indicate only that there is at least no worsening in any of the bone markers after switching to emicizumab prophylaxis (141), but additional data on the long-term effect are awaited.

More clinical and experimental studies are urgently needed because an understanding of the functional role of coagulation factors in bone health is critical to prevent and efficiently treat low BMD in patients with hemophilia in future. Now, where hemophilia treatment has improved dramatically over the past years, with safe factor replacement being widely available, the focus must turn to addressing such age-related co-morbidities and long-term complications. The mechanism of bone loss in hemophilia is thus important, because the mode of therapy may need to be tailored accordingly. For example, when the defect is due to defects in bone formation, patients may benefit from anabolic drugs, such as teriparatide, or therapies under development, such as neutralizing antibodies against Dkk-1 or sclerostin (142, 143). By contrast, if the bone phenotype primarily arises from defects in bone resorption, patients may respond better to antiresorptive agents, such as bisphosphonates or denosumab, a human monoclonal antibody to RANKL (144). The only clinical trial for the treatment of low BMD in patients with hemophilia evaluated the effect of a 12-month-long monthly oral administration of 150 mg ibandronate, a bisphosphonate, in ten adults (mean age 43.5 years) (145). Ibandronate was well-tolerated and led to a 4.7% increase in BMD in the lumbar spine, but not to significant changes in the femoral neck or total hip (145). Nevertheless, research so far strongly indicates that the bone phenotype develops already early in the life of hemophilia patients and most of the mentioned therapeutic agents should, due to their partly severe side-effects, be avoided or not frequently used in children (65, 146).

In summary, further studies are urgently needed to determine the functional role of FVIII in bone metabolism and to provide guidance for the development of effective and safe treatment strategies in order to prevent low BMD in patients with hemophilia.

## Data Source and Search

A literature search strategy was developed by consulting the PubMed platform of the National Center for Biotechnology Information (NCBI). The literature search performed included peer reviewed papers, published in English language, updated to December 2020. The search strategy used a combination of controlled key words (e.g., “coagulation,” “hemophilia,” “factor VIII,” “bone mineral density,” “osteopenia,” “osteoporosis,” and “bone metabolism”). Ninety-five references were screened for their relevance and quality. Of these, a total of 57 papers were considered and complemented by reading review articles, meta-analyses and additional literature from the respective reference sections. Based on the high number of manuscripts manually added, this review has to be considered as a scoping review with selection bias (see Supplementary Figure 1) (147).

## Author Contributions

JG, MS, and WS: drafting and refining the manuscript. JG: writing the manuscript and preparing the figures. MS, WW, and WS: critical reading of the manuscript. All authors contributed to the article and approved the submitted version.

## Funding

Funding of JG and publication of the manuscript was supported by the Medical University of Innsbruck.

## Conflict of Interest

The authors declare that the research was conducted in the absence of any commercial or financial relationships that could be construed as a potential conflict of interest.

## Publisher's Note

All claims expressed in this article are solely those of the authors and do not necessarily represent those of their affiliated organizations, or those of the publisher, the editors and the reviewers. Any product that may be evaluated in this article, or claim that may be made by its manufacturer, is not guaranteed or endorsed by the publisher.
