# Supplementary material for: Low Bone Mineral Density in Hemophiliacs
Source: Front Med (Lausanne). 2022 Feb 2;9:794456. doi: 10.3389/fmed.2022.794456 (PMC8849249; doi:10.3389/fmed.2022.794456)
Supplement: Supplementary file 1 [file Data_Sheet_1.PDF]

Supplementary Figure 1: PRISMA flow diagram for systematic reviews which includes search of database and other sources.

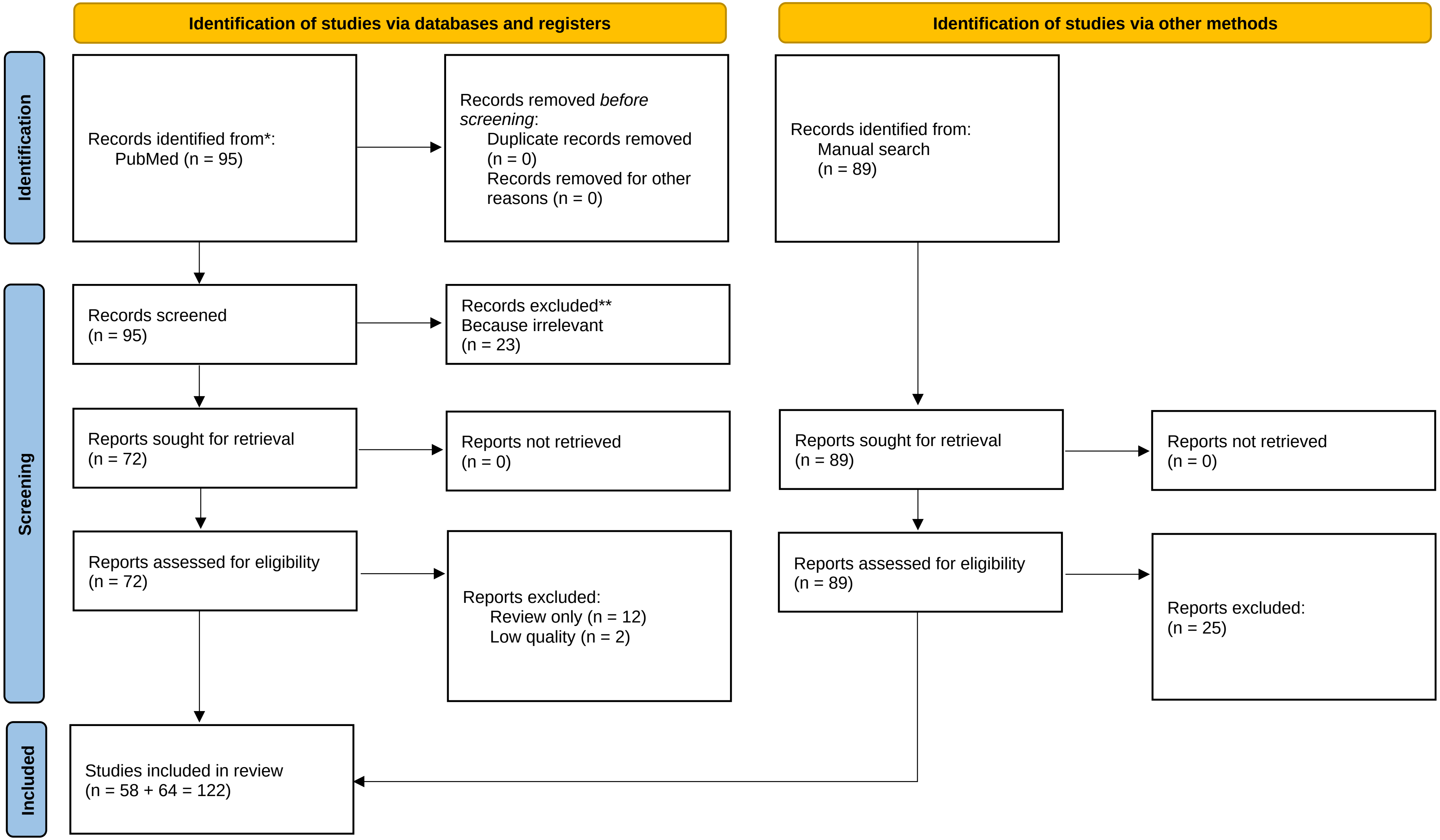

*Consider, if feasible to do so, reporting the number of records identified from each database or register searched (rather than the total number across all databases/registers).
**No automation tool was used.

From: Page MJ, McKenzie JE, Bossuyt PM, Boutron I, Hoffmann TC, Mulrow CD, et al. The PRISMA 2020 statement: an updated guideline for reporting systematic reviews. BMJ 2021;372:n71. doi: 10.1136/bmj.n71.
